# Retrospective estimate of COVID-19 infections in nine Colombian cities in 2020

**DOI:** 10.1017/S095026882610106X

**Published:** 2026-01-26

**Authors:** Jenny Márquez, David García-García, M. Isabel Vigo, César Bordehore

**Affiliations:** 1 Faculty of Engineering, Business and Agro-Environmental Sciences, University of Viña del Mar, Viña del Mar, Chile; 2 Applied Mathematics and Aerospace Engineering Department, University of Alicante, San Vicente del Raspeig, Spain; 3 Multidisciplinary Institute for the Study of the Environment “Ramon Margalef”, University of Alicante, San Vicente del Raspeig, Spain; 4 Ecology Department, University of Alicante, San Vicente del Raspeig, Spain

**Keywords:** Colombia, COVID-19, first infection, REMEDID, retrospective study

## Abstract

COVID-19 led to a pandemic in 2020, which officially arrived in Colombia on 6 March 2020. As in other parts of the world, the spread of the virus was underestimated due to the lack of diagnostic tests and follow-up protocols. The present study estimates the number of daily cases of COVID-19 infection compatible with theoretical knowledge of the disease, seroprevalence studies, and records of daily deaths due to the disease. To this end, the REMEDID (Retrospective Methodology to Estimate Daily Infections from Deaths) algorithm was applied in nine Colombian cities. On average, official records detected only around 13% of the maximum number of infected persons in the first wave, which they dated with a delay of 25 days. In addition, there was an average delay of 30 days in detecting the first cases. In particular, in Bogotá, the city with the highest number of infections in Colombia, it was observed that (1) the first infected person arrived on 26 January 2020, 40 days before the official registration; (2) the maximum peak of infections was around 6 times higher than that recorded in the official statistics; and (3) this peak was reached on 08 July 2020, 39 days before the official registration date.

## Introduction

In late 2019, the emergence of SARS-CoV-2, the causative agent of COVID-19, triggered a global health crisis unprecedented since the 1918 influenza pandemic. As of February 2024, there have been approximately 775 million cases and 7 million deaths worldwide [1]. The rapid sharing of viral sequence data, which began within weeks of virus identification, enabled the development of diagnostic tests critical to control efforts, and initiated research that led to candidate vaccines and therapies [2].

Between 30 January 2020 and 5 May 2023, COVID-19 was declared a global health emergency by the World Health Organization (WHO) [3, 4], the highest possible alert level. However, the pandemic is not over and remains a threat to public health [5]. On 30 January 2023, the 14th meeting of the International Health Regulations (IHR-2005) Emergency Committee on the COVID-19 pandemic warned of the need for long-term public health measures, such as the integration of COVID-19 surveillance into the Global Influenza Surveillance and Response System [6]. The recording of infections and deaths due to COVID-19 is key information for monitoring the evolution of the pandemic and making decisions to contain its spread. At the beginning of the pandemic, the number of infections and deaths was greatly underestimated in most parts of the world [7, 8], mainly due to the lack of diagnostic tests and protocols for a new and unexpected situation. Without testing, it was impossible to detect one-third of infections that were asymptomatic [9], and without adequate protocols, cases with compatible symptoms were not systematically counted. Monitoring the spread of the virus at the start of the pandemic was therefore generally very poor, although it was essential to implement public health measures to mitigate the impact of the virus. However, we can now conduct back-calculation studies that will help us to better understand the initial spread of the virus in order to be prepared to anticipate and prevent future outbreaks [2].

The back-calculation technique is used to estimate the evolution of new infections in the past based on indirect data obtained a posteriori. This methodology was first used in the late 1980s with HIV infections [10], a field in which it became very popular, leading to refinement of the technique as it was adapted to the evolution of the disease itself and its treatment [11, 12]. Back-calculation has also been applied to other diseases such as hepatitis C [13] or lung cancer [14]. It has also been applied in COVID-19. For example, a back-calculation analysis was applied to estimate the timing of infections from the time of diagnosis, using the probability distribution of the time between infection and diagnosis in Australia [15]. In Ireland, undiagnosed infections were estimated based on COVID-19 death data and an estimated case fatality rate (CFR) [16].

In this study, we will use a back-calculation technique that was designed for COVID-19: the REMEDID algorithm (Retrospective Methodology to Estimate Daily Infections from Deaths), which allows us to estimate daily infections from daily COVID-19 death data and seroprevalence studies that allow us to determine the total number of infected people on a given date [17]. This algorithm was originally applied in Spain and made it possible to determine that on 14 March 2020, the day on which national containment was imposed, with an official record of 1832 infections on the same day, there were in fact between 64,000 and 78,000 infections on that day, that is, 35–42 times more. One of the most important aspects to be taken into account in viral cases is the identification of the first case of infection, which is always complicated. In the United States of America (USA), the same algorithm determined that the virus was likely to have entered the country on 28 December 2019, 16 days before the first case was recorded in the country [18]. In addition, the virus arrived on average 1 month before it was detected in each state. In the Metropolitan Region of Chile, results were similar to those in Spain and the USA [19].

In Colombia, on 6 March 2020, the Ministry of Health and Social Protection confirmed the first case of COVID-19 in a 19-year-old patient traveling from Milan, Italy, who presented symptoms and went to the health services, where samples were taken and tested positive [20]. Two and a half weeks later, at midnight on 24 March 2020, a nationwide quarantine was imposed, which lasted almost 4 months until 15 July 2020. From then on, the restrictions were de-escalated in municipalities that had not registered any coronavirus cases and in populations with low infection rates [21]. On 29 December 2023, a cumulative total of 6,387,145 confirmed cases had been registered in Colombia [22].

The aim of this study is to provide information on the first COVID-19 infections in 9 cities in Colombia, based on the application of the REMEDID algorithm. The study is limited to the 9 cities where seroprevalence studies have been carried out [23]: Barranquilla, Medellín, Cúcuta, Bucaramanga, Bogotá, Cali, Villavicencio, Leticia, and Ipiales. Although a seroprevalence study was also carried out in Guapi, this city was not included in the study for reasons explained in Section title *“Data*”.

## Methodology

### REMEDID algorithm

From a methodological point of view, a retrospective study refers to a type of observational research commonly used in the health field, with the aim of looking at past events in order to carry out a chronological analysis aimed at understanding the present. As mentioned above, the retrospective method chosen for this study is REMEDID [17]. With this method, the time series of daily infections can be estimated from (1) the probability distribution measuring the time elapsed from the infection of an individual to death; (2) the daily records of COVID-19 deaths; and (3) the cumulative number of infections in a given time. As the algorithm is applied when infections have not been properly measured, cumulative infections are usually obtained from seroprevalence studies. REMEDID is useful when the recording of deaths is more reliable than that of infections, as is the case in Colombia. This is because the Colombian National Institute of Health (INS, for its acronym in Spanish) has a retrospective death analysis process in which death certificates are re-analysed to identify and correct possible errors in diagnoses [24]. These analyses consist of postmortem diagnostic tests, which are complemented by verbal autopsies, clinical probability algorithms, medical history, and epidemiological context.

The REMEDID algorithm connects the date of infection (DI) with the date of death (DD) caused by the disease using the equation

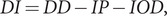

where IP refers to the incubation period and IOD to the period from illness onset to death. In other words, if we add to the date of infection the period of incubation and the period of onset of illness until death, we obtain the date of death.

The term corresponding to DD is obtained from the official records of COVID-19 deaths, while IP and IOD are values that may vary from individual to individual, so we will estimate them using probability distributions. Let *X_IP_* be the random variable (r. v.) representing the incubation period and *X_IOD_* the r. v. for the period from illness onset to death. These r. v. are defined by their Probability Density Functions (PDF), which we can denote as *g*(*x*) for *X_IP_* and *h*(*x*) for *X_IOD_.* The sum of both r. v. produces a new r. v. that we will call *X_IP + IOD_*, which represents the time from infection to death, and whose PDF, *f*(*t*), is obtained by convolution of *g*(*x*) and *h*(*x*):

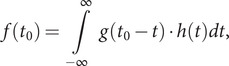

where *t_o_* is a positive real number representing the time since infection.

During the early months of the pandemic Linton et al. [25] conducted a study in Wuhan, China, and approximated *g*(*x*) by a lognormal distribution with a mean of 5.6 days (95% confidence interval, 95% CI: 5.0–6.3) and a median of 5 days (95% CI: 4.4–5.6); and *h*(*x*) by another lognormal distribution with a mean of 14.5 days (95% CI: 12.5–17.0) and a median of 13.2 days (95% CI: 11.3–15.3). The convolution of the two, *f*(*t*), does not follow a lognormal, although it is very similar (Figure 1 in [10]). *f*(*t*) has an estimated mean of 20.1 days, a median of 18.8 days and a 95% probability that *X_IP + IOD_* is less than or equal to 33 days.

**Figure 1.**
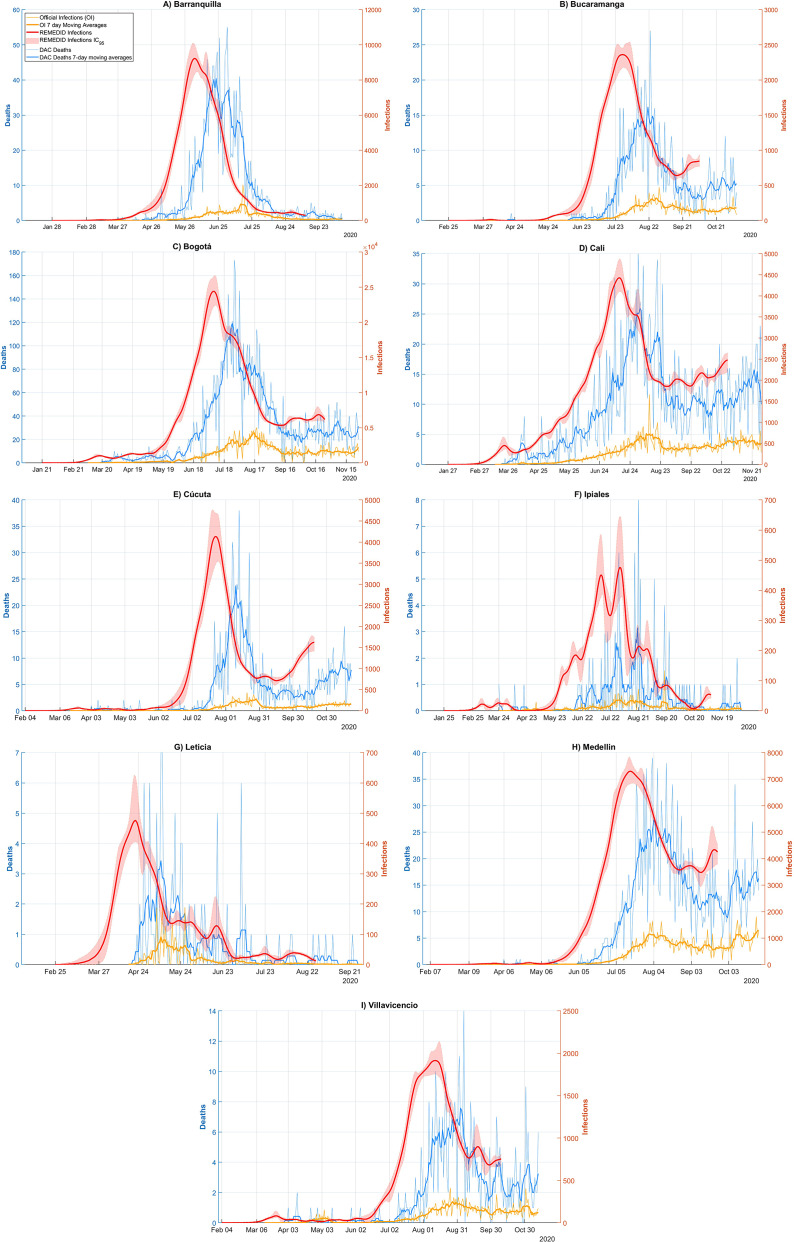
COVID-19 infections and deaths in Barranquilla, Bucaramanga, Bogotá, Cali, Cúcuta, Ipiales, Leticia, Medellín, and Villavicencio. Red line: REMEDID infections; red shade: 95% CI of REMEDID infections; yellow line: official infections; thick yellow line: 7-day moving averages of official infections; blue line: DAC deaths; thick blue line: DAC deaths 7-day moving averages.

Once the distributions have been fixed, it is possible to estimate the time series *y*(*t*) of daily infections, which yields the time series of deaths *x*(*t*), assuming a CFR equal to 100%. Infections at a given time *t_0_* are estimated by the expression:

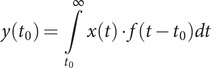



Since in our case the time series *x*(*t*) is given in days, that is, the time variable *t* is discrete and represents days, we will denote it by *x*(*n*), where *n* are integers expressing days. In order to operate with the discretized time series, we will work with *F*(*n*), the discrete version of *f*(*t*) which is obtained with the following expression:

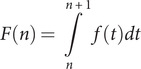



Therefore, we can now define the REMEDID algorithm by the following two equations. The first one is:
(1)

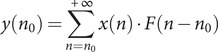



The second equation is used to adjust the estimated value of the CFR and has the following expression:
(2)

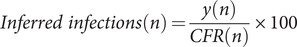



At the end of the series, we will eliminate at least 33 days, which is the upper limit of the 95% of the cumulative values of *X_IP + IOD_.* Therefore, the time series of infections will end 33 days before the time series of deaths.

### Error estimation

The PDF of IP and IOD are defined by parameters associated with confidence intervals. The distribution of errors within confidence intervals has not been specified; however, as they typically follow normal distributions, we will assume this distribution with a mean equal to the reported value of the parameter and a standard deviation equal to a quarter of the length of the interval. Uncertainty in the parameters generates uncertainty in the PDF, which will be propagated to the estimate of daily infections. To estimate the error in infections, a Monte Carlo simulation will be performed with 10,000 possible random combinations of the parameters defining the IP and IOD PDF. The REMEDID algorithm is applied to each of these PDFs, resulting in 10,000 simulations of daily infections. The 2.5 and 97.5 quartiles of the results of these simulations will define the extremes of the 95% CI. In general, the results will be presented with the mean of the simulations accompanied by the 95% CI [19].

## Data

For the cities considered in Colombia, data on deaths and daily infections were obtained from the INS from the Colombia Open Data (DAC, for its acronym in Spanish) section, which are part of the COVID-19 Positive Cases in Colombia [26].

The data considered were downloaded between 24 April and 11 May 2023. The cities studied in this research are Barranquilla, Bogotá, Bucaramanga, Cali, Cúcuta, Leticia, Medellín, and Villavicencio. The city of Guapi was not included in the study, as the calculation of the CFR value of this city produced outliers, as can be seen in Table 1.

**Table 1.** Information on the seroprevalence study carried out in the 10 cities of Colombia

City	Samples	Positive	Inhabitants per city	Infected by city	Infected (%)	Date of first official death	Deaths until the end of the study	CFR (%)
Leticia	1,417	727	42,844	21,850	51	23 April 2020	106	0,49
Medellín	1832	434	2,372,330	569,359	24	06 April 2020	1891	0,33
Barranquilla	1,487	709	1,120,103	448,041	40	27 March 2020	1713	0,38
Bucaramanga	1,429	400	528,855	148,079	28	22 April 2020	831	0,56
Cúcuta	1,453	508	629,414	220,295	35	03 April 2020	954	0,43
Villavicencio	1,457	434	451,212	135,364	30	03 April 2020	429	0,32
Bogotá	4,597	1,210	7,181,469	1,867,182	26	20 March 2020	7,922	0,42
Cali	1979	523	1,822,869	473,946	26	23 March 2020	2,431	0,51
Guapi	720	422	24.37	14,182	59	11 June 2020	7	0,05
Ipiales	1,466	445	105.517	31.655	30	24 March 2020	116	0,37
Total	17,837	5,812	14,278,650	3,929,954			16,400	0,39
Total (excluding Guapi)	17,117	5,390	14,254,613	3,915,772			16,393	0,42

*Source:* The number of infected per city is obtained by multiplying the population of the city by their positivity according to the seroprevalence study. The row ‘Total’ shows the sum of population *[31]*, infected and deaths in the 10 cities. The same for the row ‘Total (without Guapi)’ but without Guapi. It also shows the percentage of associated infections and the CFR, which was calculated by dividing the total number of deaths in the 10 cities up to the end of the study by the total number of inhabitants in the 10 cities.

The CFR for each city is calculated as the ratio of cumulative deaths to cumulative infections in each city. The cumulative infections were obtained from a seroprevalence study conducted between 21 September 2020 and 10 December 2020, in which several teams canvassed the neighbourhoods of the above cities. It should be noted that the seroprevalence study was not conducted in all cities at the same time. The timing of the study depended on whether the cities had passed the peak of the epidemic (at least the first one) and the curve began to fall [27]. The first city where it was carried out was Leticia (from 21 to 30 September); the second was Barranquilla (from 5 to 14 October); the third was Bogota (from 16 October to 27 November); Cali (from 18 October to 30 November), Medellín (from 20 to 27 October), Bucaramanga (from 29 October to 9 November), Cucuta (from 11 to 21 November), Villavicencio (from 2 to 11 November), Ipiales (from 3 to 10 December), and Guapi (Cauca from 3 to 10 December) [28]. At the time of the study, there was no serodiagnostic test endorsed by the WHO. Seroprevalence was therefore determined using a serodiagnostic test developed by the Colombian authorities and commercial tests with a sensitivity greater than 85% and a specificity equal to or greater than 90% [29].

In the nine cities (excluding Guapi), 17,117 blood samples were collected, of which 5,390 were positive for COVID-19 antibodies. This represents an average infection rate of 32.58% of the samples [30], which, extrapolated to the total population of the cities, means 3,915,772 infections. As the first seroprevalence study ended on 30 September and the last on 10 December, we assume that this value was reached in the middle of this period, that is, on 4 November. In these cities, the cumulative official number of infections up to 4 November 2020 was 552,492, so only 14.14% of infections were detected. By the end of the study, 16,393 deaths were detected across all cities, giving an average CFR of 0.42%. Table 1 shows these values for each city (including Guapi), giving the individual values of the samples collected in the seroprevalence study, the percentage of infected, the number of inhabitants [31], the estimated number of infected in each city up to the end of the seroprevalence study, the COVID-19 deaths accumulated up to that date and the CFR. This last value varies from city to city, ranging from 0.32% to 0.56%, except for Guapi, which has an excessively low value of 0.05% and has not been included in the averages.

Another important finding of the seroprevalence study was that in Guapi, the city with the highest percentage of infected people, the population lives mostly in extreme poverty, whereas in Leticia, an area that straddles two borders (Peru and Brazil), there was a large spread of the virus through the Amazon River, in addition to the peak experienced in May 2020, which would explain the high percentage of people who had been exposed to the virus [32]. In fact, in nearby cities, such as Manaus (Brazil) and Iquitos (Peru), seroprevalence was estimated at 66% and 71%, respectively [33]. On the other hand, more densely populated cities such as Bogota, Medellin, and Cali showed lower levels of infection. Of course, these cities implemented strict social distancing measures from March 2020, which may have affected transmission rates. However, despite the lower attack rates, these cities had the highest number of SARS-CoV-2 cases and deaths [34].

## Results

### Cities

The REMEDID algorithm was applied to the nine cities for which we calculated the CFR from the seroprevalence study, excluding Guapi. We will refer to the inferred daily infections as REMEDID infections (RI), in contrast to the officially recorded infections, which we will call official infections (OI). Figure 1 shows the RI and OI for the nine cities: a) Barranquilla; b) Bucaramanga; c) Bogotá; d) Cali; e) Cúcuta; f) Ipiales; g) Leticia; h) Medellín; and i) Villavicencio. The shaded area around the RI curves is the error calculated using the Monte Carlo method. Note that the RI end 33 days before the end of the seroprevalence study.

Figure 1 shows very significant differences between RI and OI. One of the most important is the date of the first infection, quantified in Table 2. Considering the average of seven of the nine cities (Leticia, Barranquilla, Cúcuta, Villavicencio, Bogotá, Cali, and Ipiales), RI start 37 days earlier than OI. In Bucaramanga, we did not find a statistically significant difference between the first REMEDID infection and the official one, since the first RI appears on 14 March 2020 (95% CI: 04 March 2020–2025 March 2020), but the date of the first OI is 15 March 2020. The time lag in the first infection means that the arrival of the first infected person in each city was generally not detected. Among all the cities, the case of Leticia stands out, because when the first OI case was officially detected, in reality there had already been a total of 5,338 RI cases, and it is also the city with the largest difference in days between the first RI infection and the OI, being 47 days (95% CI: 23–51). The second highest number of RI not detected until the first OI was observed in Bogotá with 3,534 cases. On the other hand, Ipiales is the city with the second largest difference in days between the first RI and the first OI with 42 days. For the rest of the cities, see Table 2.

**Table 2.** Dates (dd/mm/year) of first COVID-19 infections for each of the Colombian cities studied, both with REMEDID and official records. Differences between first REMEDID and official infections

City	Date 1st REMEDID infection (RI)	(95% CI)	Date first official infection (OI)	Difference in days	(95% CI) difference in days	RI accumulated up to 1st OI
Leticia	01 March 2020	26 February 2020–25 March 2020	17 April 2020	47	[23, 50]	5,338
Medellín	21 February 2020	10 February 2020–08 March 2020	09 March 2020	17	[1, 28]	47
Barranquilla	12 February 2020	01 February 2020–28 February 2020	16 March 2020	33	[9, 17]	556
Bucaramanga	14 March 2020	04 March 2020–25 March 2020	15 March 2020	1	[10, 11]	2
Cúcuta	16 February 2020	10 February 2020–06 March 2020	15 March 2020	28	[9, 34]	271
Villavicencio	14 February 2020	07 February 2020–05 March 2020	13 March 2020	28	[8, 35]	238
Bogotá	26 January 2020	21 January 2020–19 February 2020	06 March 2020	40	[16, 44]	3,534
Cali	04 February 2020	29 January 2020–24 February 2020	13 March 2020	38	[9, 18]	1887
Ipiales	11 February 2020	30 January 2020–25 February 2020	24 March 2020	42	[28, 53]	495

The first case of RI in the nine cities studied, and therefore the first day on which the virus arrived in Colombia, occurred in Bogotá on 26 January 2020 (95% CI: 21 January 2020–19 February 2020), 40 days (95% CI: 16–45) before the first officially recorded infection on 6 March 2020 [34]. The first official patient arrived in Bogotá from Milan (Italy) on 26 February 2020 and did not seek medical attention until 3 March 2020 [35]. It was not the first official case in South America, as some cases had been reported in Brazil 2 weeks earlier, and shortly after that in Ecuador and Panama [36]. As expected, local transmission was added to the imported cases. In the first 2 weeks, the number of community transmission cases (i.e., excluding imported cases, which accounted for about half) exceeded 200 [36]. The second earliest city with infection was Cali, where it was reached on 4 February 2020 (95% CI: 29 January 2020–24 February 2020). From this date until the first official infection on 13 March, there were 1887 cumulative RI cases.

The other major difference between RI and OI is observed in the peak of infections, whose date and maximum number of infections has been compiled in Table 3 for the nine cities studied. The highest maximum number of daily infections is observed in Bogotá, which is not surprising as it is the city with the largest population. Table 3 shows a maximum RI of 24,375 (95% CI: 22,462–26,867) on 08 July 2020 (95% CI: 05 July 2020–10 July 2020) and a maximum OI of 4,253 infections on 16 August 2020, that is, only 17% (95% CI: 16–19%) of the maximum daily infections were detected and ~ 5 weeks later (39 days, 95% CI: 37–42). The second city with the highest maximum number of daily infections is Barranquilla with a maximum RI of 9,221 (95% CI: 8512–10,145) on 04 June 2020 (95% CI: 02 June 2020–07 June 2020) and a maximum OI of 937 infections on 15 July 2020, that is, only 10% (95% CI: 9–11%) of the maximum was detected and ~6 weeks later (41 days, 95% CI: 38–43). On the opposite side, we have Ipiales as the city with the lowest maximum daily infections. In addition, Ipiales has two infection peaks, as shown in Figure 1(F). The first peak of RI has a value of 452 (95% CI: 404–607) on 12 July 2020 (95% CI: 10 July 2020–16 July 2020) and a maximum OI of 35 infections on 30 July 2020 with a difference of 18 days (95% CI: 14–20), while the second peak with a maximum RI of 477 (95% CI: 382–663) on 01 August 2020 (95% CI: 30 July 2020–04 August 2020) and a maximum OI of 36 infections on 15 August 2020 with a difference of 14 days (95% CI: 11–16). In both peaks, only 8% (95% CI: 5–9%) of the maximum was detected. The city with the lowest detection rate of the magnitude of the first wave was Cúcuta, with a maximum RI of 4,139 (95% CI: 3564–4,803 on 23 July 2020 (95% CI: 20 July 2020–26 July 2020) and a maximum OI of 265 infections on 28 August 2020, that is, only 6% (95% CI: 6–7%) of the maximum was detected and ~5 weeks later (36 days, 95% CI: 33–39).

**Table 3.** Maximum number of RI and OI infections together with the dates on which these maximum values occurred (date format is dd/mm/year) and the difference in days between the maximum RI and OI. Ipiales has two peaks, and the confidence interval is shared between them

City	Date of maximum REMEDID infection (RI)	(CI: 95%)	Maximum infection number (RI)	(CI: 95%)	Official infection (OI) maximum date	Maximum infection number (OI)	Difference days between RI and OI maxima	CI (95%) of difference in days
Leticia	22 April 2020	20 April 2020–24 April 2020	476	405–647	10 May 2020	91	18	[16, 20]
Medellín	16 July 2020	15 July 2020–19 July 2020	7,302	6,862–7,900	31 July 2020	1,149	15	[12, 16]
Barranquilla	04 June 2020	02 June 2020–07 June 2020	9,221	8,512–10,145	15 July 2020	937	41	[38, 43]
Bucaramanga	30 July 2020	27 July 2020–05 August 2020	2,363	2,184–2,568	29 August 2020	318	30	[24, 33]
Cúcuta	23 July 2020	20 July 2020–26 July 2020	4,139	3,564–4,803	28 August 2020	265	36	[33, 39]
Villavicencio	11 August 2020	09 August 2020–16 August 2020	1916	1759–2,165	27 August 2020	249	16	[11, 18]
Bogotá	08 July 2020	05 July 2020–10 July 2020	24,375	22,462–26,867	16 August 2020	4,253	39	[37, 42]
Cali	13 July 2020	11 July 2020–15 July 2020	4,424	4,103–4,913	09 August 2020	730	27	[25, 29]
Ipiales	12 July 2020	10 July 2020–16 July 2020	452	404–607	30 July 2020	35	18	[14, 20]
Ipiales	01 August 2020	30 July 2020–04 August 2020	477	382–663	15 August 2020	36	14	[11, 16]

### Colombia

Since we do not have a national seroprevalence study in Colombia, we will estimate the CFR needed to apply REMEDID as the ratio between deaths and total infections in the nine cities with a seroprevalence study, assuming that the average value of the nine cities is the same for all of Colombia, a CFR value of 0.42% is considered. This value is only an assumption that cannot be verified, so the validity of the results below depends on the accuracy of this assumption.

**Figure 2.**
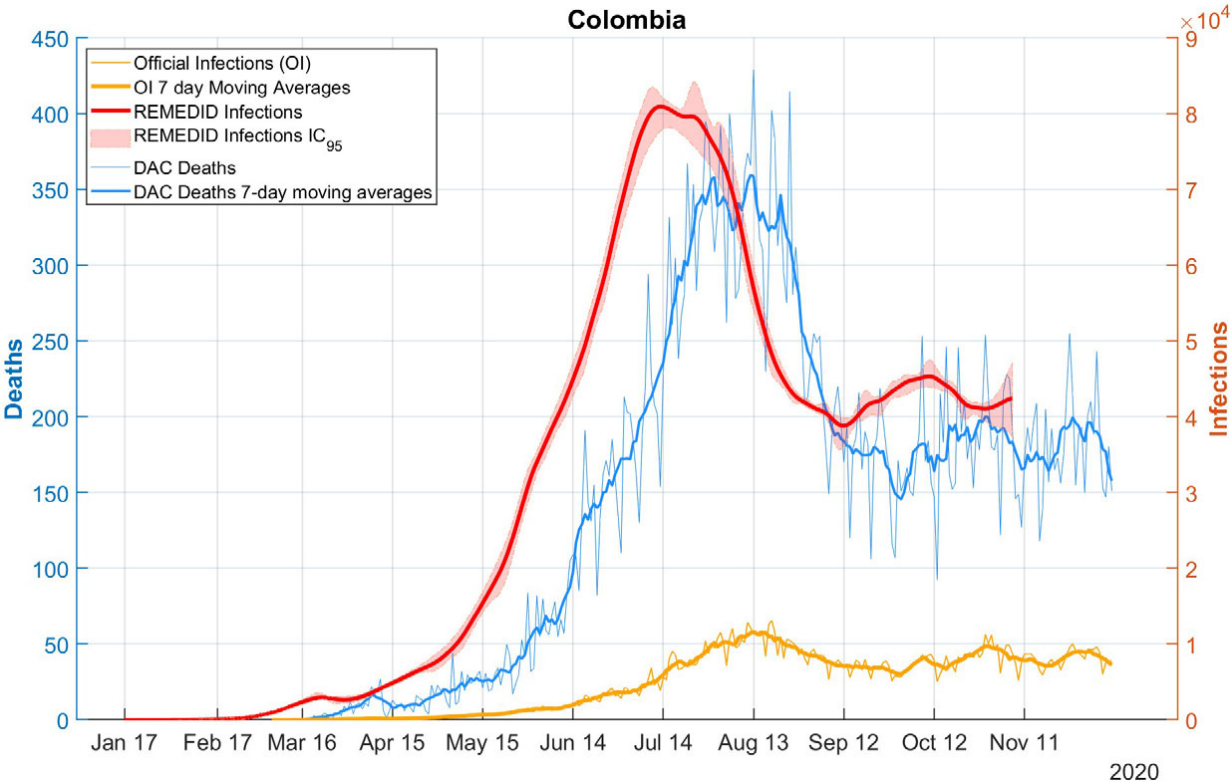
COVID-19 infections and deaths in Colombia. Red line: REMEDID infections; red shade: 95% CI of REMEDID infections; yellow line: official infections; thick yellow line: 7-day moving average of official infections; blue line: DAC deaths; thick blue line: DAC deaths, 7-day moving averages.

Figure 2 shows the RI time series estimated for the whole of Colombia, assuming the aforementioned CFR of 0.42%, based on daily DAC deaths. Comparing the RI and OI series, similar differences to those discussed for the nine cities are observed: i) the date of first infection is 24 January 2020 (CI: 21 January 2020–16 February 2020) according to REMEDID, which is 42 days (95% CI: 12–49) before the first officially recorded infection on 6 March 2020; ii) the peak of RI during the first wave is 80,916 (95% CI: 78,126–84,595) and is reached on 13 July 2020 (95% CI: 11 July 2020–25 July 2020), while the peak of the OI is reached 32 days later on 14 August 2020 with 11,928 infections; and iii) the official data present 7-day cycles (the date and value of the maximum have been calculated using 7-day moving averages).

At the national level, as in the cities studied, there was an underestimation of official infections with a total of 7,544 cumulative RI in Colombia up to the date of the first OI and a total of 1,406,305 official infections up to the end of the seroprevalence study, while the RI were 9,121,258 (95% CI: 8,792,689–9,294,904). This means that on average 15.42% (95% CI: 15.13%–15.99%) of infections were detected.

## Discussion

The REMEDID algorithm has been applied in 9 cities in Colombia, allowing realistic estimates of the number of daily COVID-19 infections for the period January–September 2020, and the new results reconcile the discrepancies between: (i) available stochastic information on COVID-19, such as IP and IOD distributions; (ii) seroprevalence studies, which provide a realistic total number of infections; and (iii) daily mortality time series [18].

This new information allows us to better understand the spread of the virus in Colombia during the initial phase of the pandemic. The new findings can be summarized in three points: (1) in general, the arrival of the virus in the nine cities studied in Colombia was known very late, with an average delay of about 30 days. This probably facilitated the spread of the virus, since the lack of warning reduces the motivation to apply containment measures that do not seem so necessary; (2) the peak of daily infections was underestimated, with only 6% to 19% of the actual infections detected. This underestimation led the Colombian population to take fewer precautions than they would have if they had been aware of the true severity of the situation; (3) the number of daily infections began to fall well before this reduction was observed, about 25 days on average. This delay was detrimental in the sense that the measures adopted did not appear to be effective, and citizens were discouraged from applying them.

It should be noted that a CFR has been calculated for each city and for the country as a whole, which is constant over the study period, which ends in October 2020 at the latest. There are two major factors that influenced the CFR. The first was the different variants of the virus. The first variant, B.1.1.7 lineage (also known as the alpha variant), appeared in England in September 2020 and spread from there around the world, but not quickly enough to affect our study. It should be noted that this variant accounted for 30% of infections in Spain in January 2021. You can find a summary of the timeline in [37]. The second factor that influenced the CFR was vaccines, but these were not administered during the study period. Finally, the saturation of hospital beds and ICUs (intensive care units) may have had a temporary influence on the CFR, but in the case of Colombia, there was no such saturation [38, 39]. Therefore, our assumption that the CFR remained constant until October 2020 seems reasonable.

The lack of reliable information on the number of daily infections is not unique to Colombia; similar situations have been confirmed in Spain [17], the USA [18], and Chile [19], where the REMEDID algorithm was also applied. Furthermore, the underestimation of infections was a widespread feeling, confirming the words of the Minister of Health, Mr. Fernando Ruiz, who said that ‘many people in Colombia could have had COVID and have been asymptomatic; other people could have had the disease, had symptoms and not consulted, so they were never registered as having had COVID’ [36]. In fact, seroprevalence studies were conducted to know the true extent of the pandemic. The added value of the REMEDID algorithm is the temporal ordering of the infections detected in these seroprevalence studies.

Determining the initial case of a pandemic is a challenging process, a fact that has been further highlighted in the context of the pandemic of COVID-19 due to the high proportion of asymptomatic and mildly symptomatic individuals, as well as underreported cases [9, 40–46]. The results presented here suggest that the virus was circulating in Colombia for approximately 1 month prior to its detection. This finding may be regarded as surprising, however, a number of studies employing diverse methodologies have documented analogous cases in several countries. The initial report of the virus was on 31 December 2019, when the Wuhan Municipal Health first reported a cluster of pneumonia cases of unknown origin [47]. This was the first time that official news about the SARS-CoV-2 was reported, before it was given a name. It is assumed that the virus then spread throughout the world. However, retrospective studies using different techniques suggest that the virus arrived much earlier than the official detection dates in various parts of the world. For example, in France, retrospective testing of respiratory specimens from a patient hospitalized on 27 December 2019, yielded a positive result for SARS-CoV-2, approximately 1 month prior to the first officially reported case in the country [48]. In Italy, a retrospective analysis of wastewater samples revealed the presence of the virus in Milan and Turin as early as 18 December 2019 [49]. Moreover, a retrospective computational analysis indicated that the initial infection in Italy occurred in late November 2019 [50]. In the USA, retrospective analysis of blood samples has led to the identification of virus introduction at earlier points in time than previously reported in Illinois, Massachusetts, Wisconsin, Pennsylvania, and Mississippi [51], and even between December 13–16, 2019, in California, Oregon, and Washington [41]. The findings of numerous retrospective studies indicate that the virus had already disseminated across multiple countries within a timeframe ranging from 1 month to several days prior to the official statement released by the Wuhan authorities. The findings of this study are based on a distinct methodology; nevertheless, the results are consistent with those of earlier studies.

In Colombia, a state of emergency was declared on 17 March 2020, and as of 25 March 2020, extended until 15 July 2020, mandatory preventive isolation or quarantine of all inhabitants of the Republic of Colombia was declared. From 16 March 2020, no travellers were allowed to enter Colombia, with the exception of Colombian nationals, foreigners residing in the country and diplomats. International flights were not allowed until the end of May, except for cargo and humanitarian reasons. Domestic flights would not be allowed until the end of the sanitary emergency, except for specific cases of health, public order, humanitarian, and government services [52], all of which are similar to the measures considered by other countries, such as those in Europe [53], Africa [54], Asia [55], and Indonesia [56].

Despite the fact that quite strong containment measures were already in place in Colombia in March 2020, infections continued to rise until July in most of the cities studied. This suggests that the population may not have followed the measures with the necessary rigor until the situation became more complicated with the increase in deaths of friends and family members. However, with the information we have, this is pure speculation.

## Data Availability

The data and software used for the study are available on the GitHub repository: https://github.com/dgarciaUA/epidemiology.
